# Modification of Activated Carbon and Its Application in Selective Hydrogenation of Naphthalene

**DOI:** 10.1021/acsomega.2c03914

**Published:** 2022-10-17

**Authors:** Zhenhui Liang, Shaoqing Guo, Hongyu Dong, Zhenrong Li, Xing Liu, Xin Li, Hefei Kang, Li Zhang, Lijing Yuan, Liangfu Zhao

**Affiliations:** † Institute of Coal Chemistry, Chinese Academy of Sciences, Taiyuan030001, China; ‡ University of Chinese Academy of Sciences, Beijing100049, China; § Taiyuan University of Science and Technology, Taiyuan030024, China

## Abstract

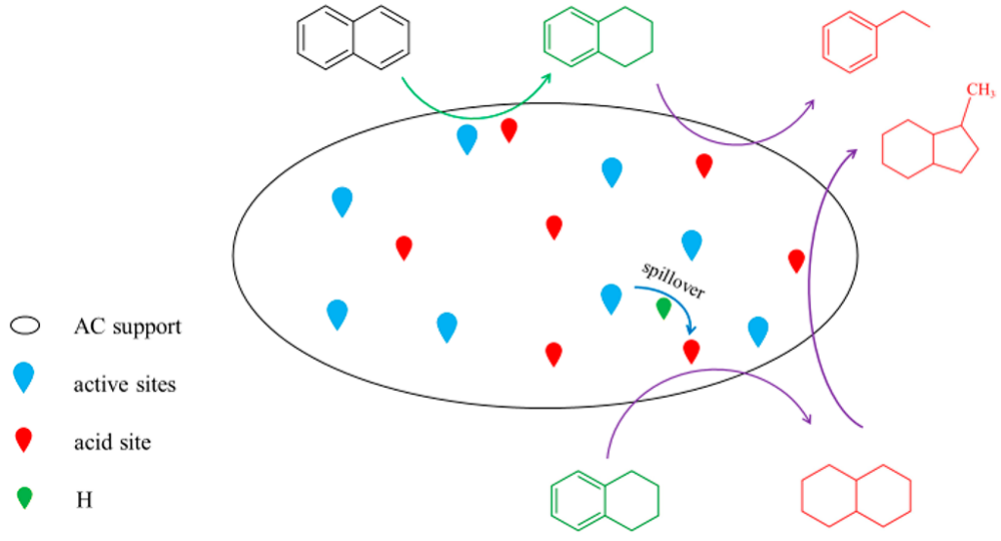

The MoS_2_/ACx catalyst for hydrogenation of naphthalene to tetralin was prepared with untreated and modified activated carbon (ACx) as support and characterized by X-ray powder diffraction, Brunauer–Emmett–Teller, scanning electron microscopy, temperature-programmed desorption of ammonia, X-ray photoelectron spectroscopy, and scaning transmission electron microscopy. The results show that the modification of activated carbon by HNO_3_ changes the physical and chemical properties of activated carbon (AC), which mainly increases the micropore surface area of AC from 1091 to 1209 m^2^/g, increases the micropore volume of AC from 0.444 to 0.487 cm^3^/g, increases the oxygen-containing functional groups of AC from 5.46 to 7.52, and increases the acidity of catalysts from 365.7 to 559.2 mmol/g. The modified catalyst showed good catalytic performance, and the appropriate HNO_3_ concentration is very important for the modified of activated carbon. Among all the catalysts used in this study, the MoS_2_/AC3 catalyst could achieve the highest yield of tetralin. It can be attributed to the moderate acidity of the catalyst, reducing the cracking of hydrogenation products. Also, the proper hydrogenation activity of MoS_2_ and the appropriate increase of oxygen-containing functional groups on the surface of modified activated carbon are beneficial to the dispersion of active components on the support, increasing the yield of tetralin. The catalytic performance of MoS_2_/AC3 is better than that of MoS_2_/Al_2_O_3_ catalyst, and the two catalysts show different hydrogenation paths of naphthalene.

## 1 Introduction

Polycyclic aromatic hydrocarbons (PAHs) make up an important pollutant that is harmful to the ecological environment,^1−6^ and they are also an important raw material for high value-added products.^7−9^ Naphthalene is the most abundant PAH in coal tar with a content of 8–12%, and naphthalene can be converted into tetralin and decalin through hydrogenation treatment.^10,11^ Compared with naphthalene and decalin, tetralin has higher industrial value. As an ideal high boiling point solvent, tetralin is widely used in medicine, paint, papermaking, coatings, agrochemical, and other fields. As a hydrogen storage material, the hydrogen production rate is 3.9–6.3-times that of decalin under the same conditions, which has incomparable advantages and application prospects compared with other hydrogen storage materials.^10−14^ Therefore, the selective hydrogenation of naphthalene into tetralin is of great significance.

The catalytic hydrogenation of naphthalene is a complex reaction with hydrogenation saturation, cracking, and isomerization. In order to obtain tetralin, the selective hydrogenation catalyst is important. Also, the active components and supports are important for the selective hydrogenation catalyst. The catalysts with precious metal have the advantages of high activity under low reaction temperature and pressure, but their high cost as well as the sensitivity to sulfur and nitrogen hinders their wide application in industry.^10−13,15,16^ Generally, transition metal catalysts supported on Al_2_O_3_, HY molecular sieves, and Al_2_O_3_-ZSM-5 have been used as catalysts for naphthalene hydrogenation to tetralin.^12,17,18^ For example, MoP/HY catalyst was used for catalytic hydrogenation of naphthalene to tetralin at 300 °C and 4 MPa, but the yield of tetralin was only about 82%.^17^ The bimetallic Ni–Mo/Al_2_O_3_ catalyst is also used for catalytic hydrogenation of naphthalene to tetralin and the yield of tetralin can reach more than 90%, but the reaction needs to be carried out under higher pressure of 6 MPa.^12^ Therefore, it is necessary to develop a new catalyst with a low cost and energy consumption as well as a high the yield of tetralin.

Activated carbon has great potential to be used as carrier of the catalyst because of its large specific surface area, developed pore structure, strong adsorption capacity, acid and alkali resistance, and easily adjustable surface properties.^10,13,19−22^ For example, the Mo_2_C/AC catalyst used by Pang et al. can achieve a tetralin yield of 88.4% at 340 °C and 4 MPa.^13^ The MoP/AC catalyst prepared by Usman et al. could achieve 82% conversion of naphthalene and 81% yield of tetralin at 300 °C and 4 MPa.^10^ However, the chemical inertness of carbon might result in a low reactivity because of the interaction between the activated carbon and the metal precursors supported, which impacts the dispersion of the metals significantly.^19^ It is reported that the modification of activated carbon has a very important effect on the dispersion of supported metal particles and the performance of the catalyst.^19,21^ The surface modification of activated carbon mainly changes its chemical properties due to the introduction of surface functional groups, and then its physical properties such as specific surface area and pore volume are also changed.^19^ On one hand, the functional groups introduced can improve the surface hydrophilicity of activated carbon and make the metal precursor solution enter the pore of the carrier more easily during the impregnation stage so that the metal dispersed more uniform. On the other hand, the functional groups can be used as anchor of metal to improve the dispersion of active metals and then change the activity of the catalyst and the selectivity of the product.^19,22^ The commonly used method for the modification of activated carbon is HNO_3_ treatment. The HNO_3_ treatment can introduce surface functional groups into the surface of activated carbon, thus improving the dispersion of active components on activated carbon and significantly improving the catalytic performance of the catalysts.^20,22,23^

In this paper, the MoS_2_/ACx catalysts were prepared with activated carbon treated by HNO_3_ as support. The effects of different concentrations of HNO_3_ treatment on the surface properties and the dispersion of active mental on activated carbon were studied. The performance of the MoS_2_/ACx catalyst was investigated in the selective hydrogenation of naphthalene to tetralin. For comparison, the performance of the MoS_2_/Al_2_O_3_ catalyst was also investigated in the same reaction.

## 2 Experimental Section

### 2.1 Modification of Activated Carbon and Preparation of Catalyst

The 6 mL nitric acid solution with concentration of 1, 2, 3, and 4 mol/L was placed in a beaker containing 3 g of activated carbon (AC, Shanghai Macklin Biochemical Co. Ltd.). AC was oxidized by HNO_3_ at room temperature for 6 h, then filtered, washed to neutral, and dried at 80 °C. The samples were marked as AC1, AC2, AC3, and AC4, respectively.

The catalyst was prepared by isovolumetric impregnation method. The carriers including ACx and Al_2_O_3_ were impregnated with an aqueous solution of (NH_4_)_6_Mo_7_O_24_·4H_2_O (0.12 g/mL) for 12 h, then dried at 120 °C for 12 h and calcined at 500 °C for 4 h under nitrogen flow. The catalysts were identified as MoO_3_/AC, MoO_3_/AC1, MoO_3_/AC2, MoO_3_/AC3, MoO_3_/AC4, and MoO_3_/Al_2_O_3_, respectively. The amount of MoO_3_ loading was 20 wt %. The catalyst obtained with particle size of 10–20 mesh was placed in the constant temperature zone of a fixed bed reactor tube for sulfidation. The catalysts were presulfided in a 5 wt % CS_2_/cyclohexane stream for 11 h by temperature programming method to obtain the sulfide catalyst under the condition of 320 °C, 4 MPa, and H_2_/oil volumetric ratio of 1000:1. The catalysts were identified as MoS_2_/AC, MoS_2_/AC1, MoS_2_/AC2, MoS_2_/AC3, MoS_2_/AC4, and MoS_2_/Al_2_O_3_, respectively.

### 2.2 Catalyst Characterization

The specific surface area and pore parameters of the support were measured by Tristar II (3020) N_2_ physical adsorption instrument. Before the test, the sample was dehydrated under vacuum at 150 °C for 12 h. The N_2_ adsorption isotherm was determined at 77 K. The specific surface area, pore volume, and average pore radius of the support were calculated by t-method according to the adsorption isotherm.

The X-ray powder diffraction (XRD) pattern was obtained with D/max2500 X-ray diffractometer. The scanning range of large angle was 10–90°, and the continuous scanning speed was 2°/ min.

The surface morphology of the catalyst was characterized by JSM-7001F thermal field emission scanning electron microscope (SEM).

The acidity of the catalyst was determined by temperature-programmed desorption of ammonia (NH_3_-TPD) using a Micrometrics Autochef II 2920. About 100 mg samples were placed in the sample rack and purged with He for 30 min at 200 °C. Then NH_3_ was adsorbed for 15 min at 100 °C and switched to He to keep 30 min. When the baseline was stable, the temperature was raised to 750 °C with 10°/min under He flow of 30 mL/min.

The dispersion of metal on the support as well as the size of metal particles in the catalyst sample was characterized by JEM-2100F field scanning transmission electron microscopy (STEM).

X-ray photoelectron spectroscopy (XPS) is a highly sensitive surface analysis technique that analyzes the chemical composition of the sample surface and the different valence states of the element, which is carried out on CEMUP with VG Scientific ESCALAB 200A spectrometer and nonmonochromatic Mg Kα radiation.

### 2.3 Catalytic Hydrogenation Test

The hydrogenation of naphthalene was carried out in a fixed bed reactor (inner diameter 10 mm). After sulfidation of the catalysts, the temperature was directly switched to the reaction temperature. Under the hydrogen pressure of 4 MPa, the cyclohexane solution of naphthalene with 5 wt % was input in the reactor by double-plunger high performance liquid phase infusion pump. The inlet flow rate was 0.04 mL/min, and the hydrogen inlet flow rate was 40 mL/min. After the reaction, the reaction products were sampled and analyzed by gas chromatography-mass spectrometry (GC-MS). The naphthalene conversion (X) and the tetralin selectivity (S) can be calculated according to the GC peak area as follows:
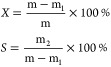
where m, m_1_, and m_2_ represent the total amount of naphthalene, the amount of remaining naphthalene, and the amount of tetralin, respectively.

## 3 Results and Discussion

### 3.1 Characterization of Activated Carbon

Table 1 lists the physical parameters (*S*_total_, S_micro_, V_micro_, *V*_total_, D) of activated carbon treated in different HNO_3_ concentrations as well as untreated activated carbon. The physical parameters of Al_2_O_3_ are also listed in Table 1. It can be seen that the HNO_3_ treatment caused the decrease of the total volume and the increase of micropore volume and micropore specific surface area. However, the HNO_3_ treatment has little effect on the average pore size of activated carbon since the average pore size of the AC1–AC4 is similar, which is consistent with the report of Li et al.^24^ Also, it shows that the support of Al_2_O_3_ has smaller specific surface area, pore volume and larger average pore size than that of activated carbon.

**Table 1:** Physical Parameters of Activated Carbon Treated with Different Concentrations of HNO_3_

	*S*_total_(m^2^ g^–1^)	*S*_micro_^a^(m^2^ g^–1^)	*V*_micro_^a^(cm^3^ g^–1^)	*V*_total_(cm^3^ g^–1^)	*D*^b^(nm)
AC	1609	1093	0.444	0.748	3.30
AC1	1499	1091	0.444	0.672	3.14
AC2	1514	1181	0.474	0.675	3.32
AC3	1488	1141	0.467	0.675	3.24
AC4	1472	1209	0.487	0.643	3.04
Al_2_O_3_	301	38	0.016	0.641	6.60

a Surface area and pore volume of micropores were calculated by the t-plot method.

b Average pore diameter was determined by the BJH method.

**Figure 1:**
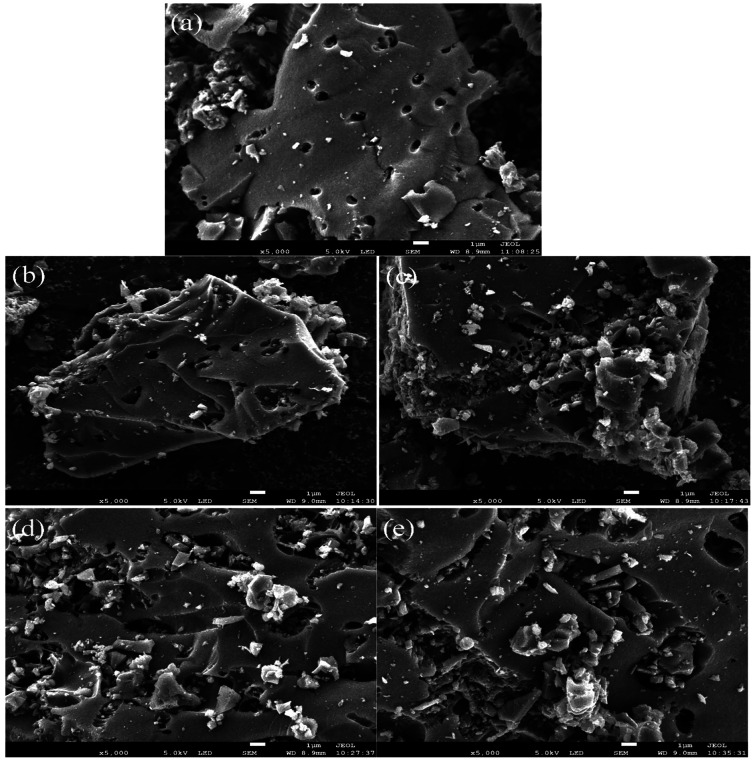
SEM images of (a) AC, (b) AC1, (c) AC2, (d) AC3, and (e) AC4.

The SEM images of ACx are shown in Figure 1a–e, respectively. Compared with the untreated activated carbon, the pore walls of the activated carbon treated with HNO_3_ have collapsed to some extent.^25^ This is consistent with the *S*_total_ results of BET characterization.

### 3.2 Characterization of Catalysts

#### 3.2.1 NH_3_-TPD of the Catalysts

**Figure 2:**
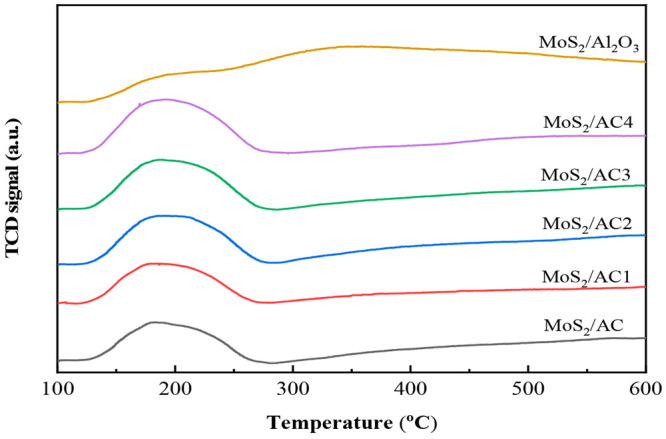
NH_3_-TPD of the catalysts.

The type and the number of acidic sites on the catalyst were determined by NH_3_-TPD, and the result is shown in Figure 2. According to the maximum desorption temperature (*T*_m_) of NH_3_ on the catalyst surface, the acid sites can be divided into weak acid sites (*T*_m_ < 200 °C), medium acid sites (200 °C < *T*_m_ < 400 °C), and strong acid sites (*T*_m_ > 400 °C).^11^ There is no peak at *T*_m_ > 400 °C for the untreated and modified catalysts, indicating that there is no increase in strong acid sites for the catalysts after HNO_3_ treating AC. This is beneficial to the reaction, which can reduce the cracking of hydrogenation products. The medium-weak acid sites increase to a certain extent, which is the result of HNO_3_ modification. The result of NH_3_-TPD for MoS_2_/Al_2_O_3_ catalyst also is shown in Figure 2, which shows peaks at *T*_m_ > 400 °C and *T*_m_ < 400 °C, indicating that the catalyst has strong acid sites and medium-weak acid sites.

Table 2 shows the amount of different acid sites of all the catalysts. Compared with the medium-weak acid site of MoS_2_/AC catalyst, the medium-weak acid site of the modified catalyst increases to a certain extent, which is beneficial to the partial hydrogenation of naphthalene, reducing the cracking of the hydrogenation product.^11^ Meanwhile, the amount of acid site for modified catalyst is lower than that of acid site for the MoS_2_/Al_2_O_3_ catalyst. Actually, the acid site also can be used as the active site.^11^ Therefore, it is probably that the hydrogenation activity of the modified catalyst may be lower than that of MoS_2_/Al_2_O_3_ catalyst since the acid site of the modified catalyst is smaller than that of the MoS_2_/Al_2_O_3_ catalyst.

**Table 2:** Quantitates of Different Acid Sites of Catalysts

catalyst	weak acidic sites (mmol/g)	moderate acidic sites (mmol/g)	strong acidic sites (mmol/g)	total acidity (mmol/g)
MoS_2_/AC	178.9	186.8	0	365.7
MoS_2_/AC1	192.9	197.3	0	390.2
MoS_2_/AC2	244.4	247.5	0	491.9
MoS_2_/AC3	247.8	259.6	0	507.4
MoS_2_/AC4	271.1	288.1	0	559.2
MoS_2_/Al_2_O_3_	102.6	832.8	209.6	1145

#### 3.2.2 XRD of the Catalysts

**Figure 3:**
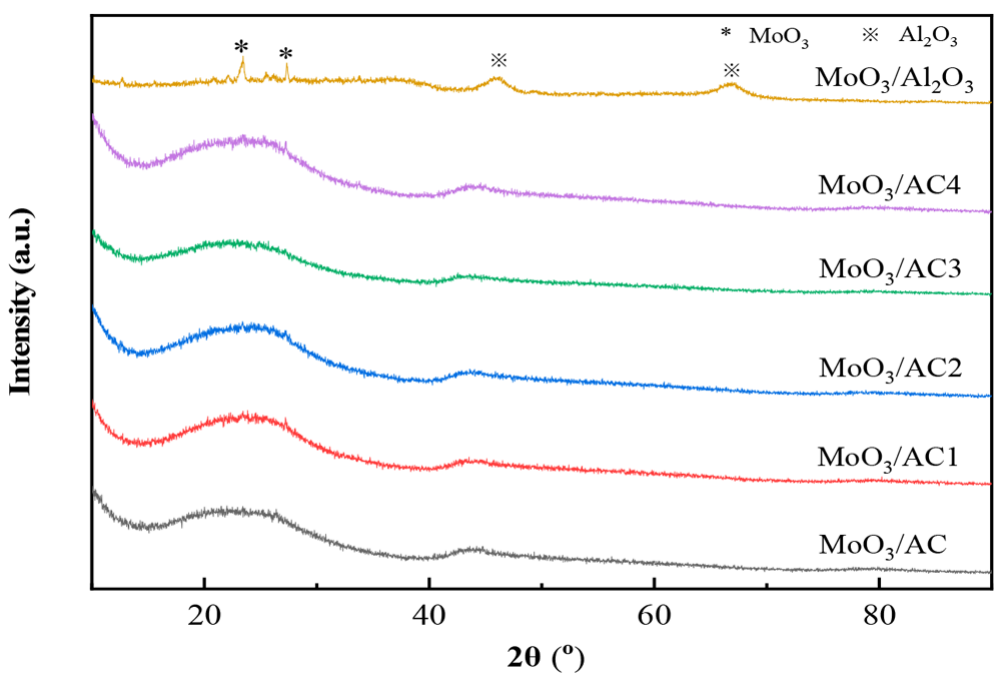
XRD patterns of different oxidation catalysts.

The result of XRD for all the catalysts is shown in Figure 3. The broad diffraction peak at 2θ 23° and 42° for catalysts supported on AC reveals a predominantly amorphous structure of AC. The characteristic diffraction peaks of oxidized metal MoO_3_ did not appear in the catalysts of ACx, which is attributed to the uniform dispersion of active components on the support. The characteristic peaks of Al_2_O_3_ (2θ = 46°, 67°) and MoO_3_ (2θ = 23°, 27°) appear in the spectra of MoO_3_/Al_2_O_3_ catalyst. This may be caused by the low dispersion of MoO_3_ on Al_2_O_3_ support.^26^

#### 3.2.3 STEM of the Catalysts

**Figure 4:**
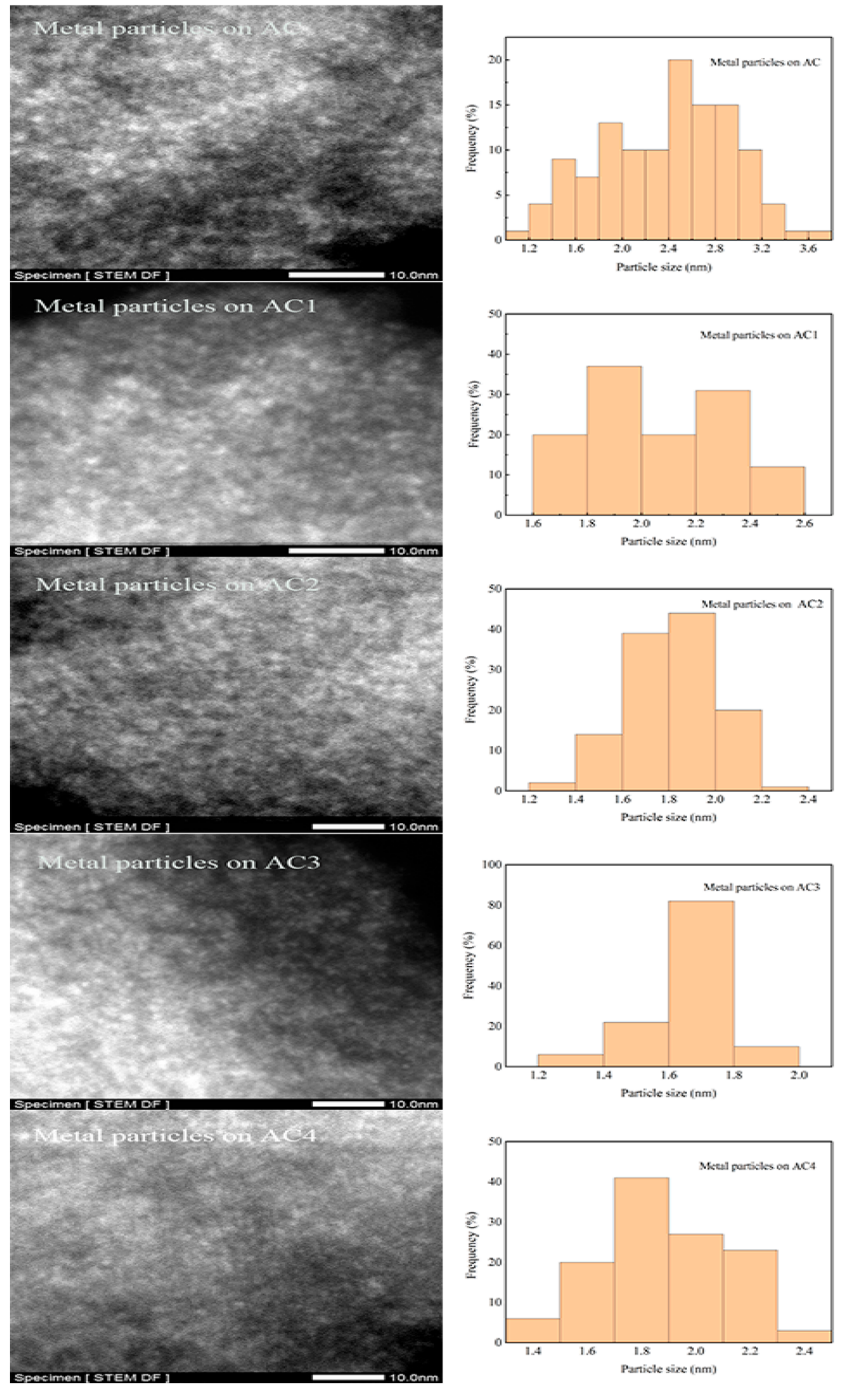
STEM images and particle size distribution of catalysts (AC–AC4).

Figure 4 shows the STEM and the particle size distribution of active metal on the catalysts of ACx. The average particle size of active metal particles was counted according to formula 1 by STEM diagram and Nano Measurer software. The dispersion of active metal was calculated by formula 2.^27^ The results are shown in Table 3 and Figure 4:
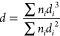
1where *d* (nm) is the average particle size and *n* is the number of the same particle size:
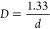
2

**Table 3:** Particle Size and Dispersion of Active Metal on the Catalysts of ACx

	*d* (nm)	*D*
AC	2.37 ± 0.1	0.56
AC1	2.05 ± 0.08	0.65
AC2	1.81 ± 0.04	0.73
AC3	1.66 ± 0.03	0.80
AC4	1.88 ± 0.05	0.71

Table 3 shows that the active metal particle size of the modified catalyst is less than that of the catalyst of AC. Meanwhile, it decreases first and then increases as the HNO_3_ concentration increases. The particle size of active metal on AC3 is the smallest. This is possibly caused by that the number of oxygen-containing functional groups introduced into the surface of activated carbon increases as the HNO_3_ concentration increases. Thus, the metal dispersion increases and the particle size becomes smaller. However, the particle size of active metal loaded on AC4 becomes larger again, which is due to the accumulation of too many oxygen-containing functional groups on activated carbon, resulting in enhanced interaction between oxygen-containing functional groups and active metal and weakening the dispersion of active metal. The active metal particle size supported on AC3 is the smallest, indicating the best dispersion of the active metal particles in the catalyst. Therefore, it can be inferred that the MoS_2_/AC3 may present good catalytic performance.

#### 3.2.4 XPS of the Supports and the Catalysts

**Figure 5:**
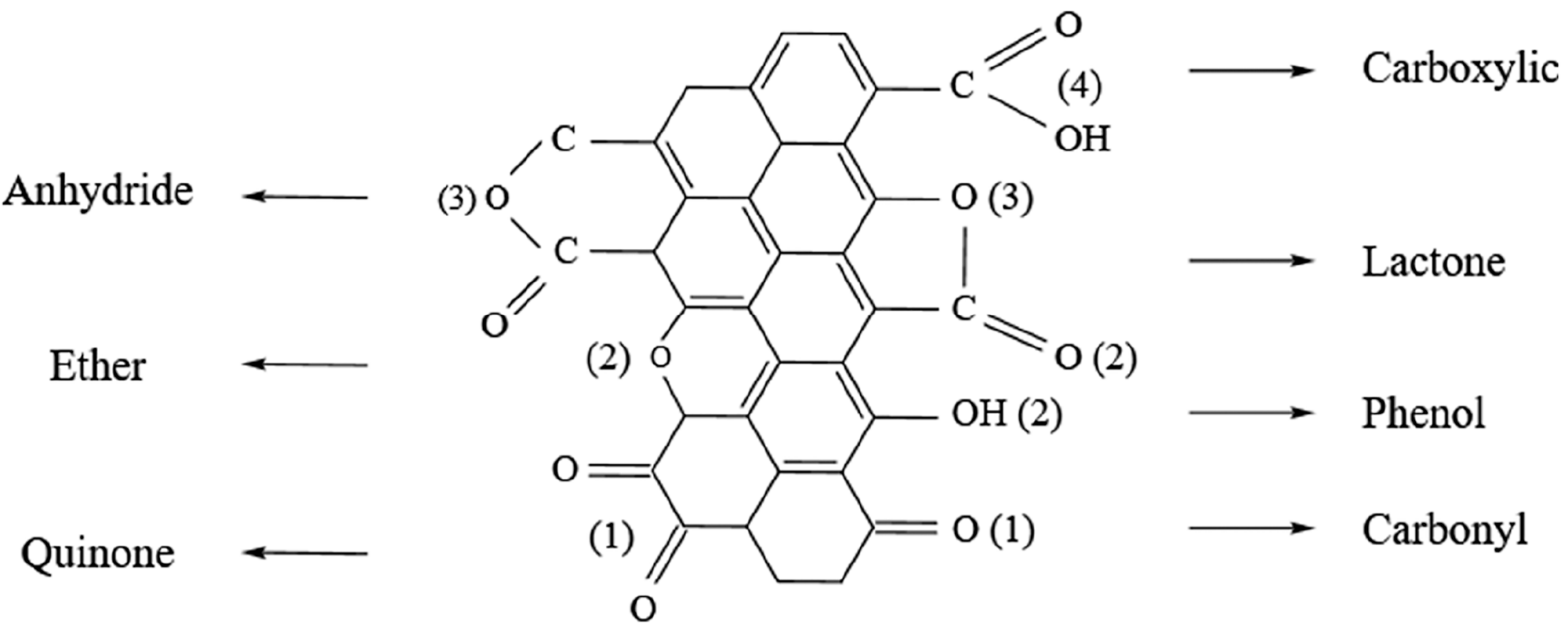
Surface functional groups on carbon.^29^

The concentrations of oxygen on the surface of activated carbon can be calculated by XPS.^28,29^ In addition, different kinds of surface functional groups on the surface of the activated carbon can also be detected by XPS. The reconstruction of the O 1s peak give information on the nature of the surface oxygen-containing functional groups.^28,29^Table 4 shows the results of XPS for the O 1s region. The carbonyl and quinone, phenol and ether, lactone and anhydride, as well as carboxylic shown in Figure 5 will correspond to the peaks located at binding Energy of 531.1 eV (1), 532.3 eV (2), 533.3 eV (3), and 534.2 eV (4), respectively.^28^ It can be seen from Table 4 that the quantity of oxygen-containing functional groups (*O*_total_) increase with the increase of HNO_3_ concentration. The increase of oxygen-containing functional groups may improve the dispersion of active components on the support and then affect the catalytic performance of the catalyst.^20^

**Table 4:** XPS Results for the O 1s Region, Values Given in % of the Total Amount

	binding energy (eV)				
sample	531.1^a^	532.3^a^	533.3^a^	534.2^a^	*O*_total_
AC	0.67	2.22	1.91	0.66	5.46
AC1	0.74	2.72	2.36	0.74	6.56
AC2	0.76	2.92	2.52	0.77	6.97
AC3	0.76	3.00	2.58	0.82	7.16
AC4	0.77	3.21	2.62	0.92	7.52

a Oxygen atoms of the relevant surface functional groups as shown in Figure 5.

**Figure 6:**
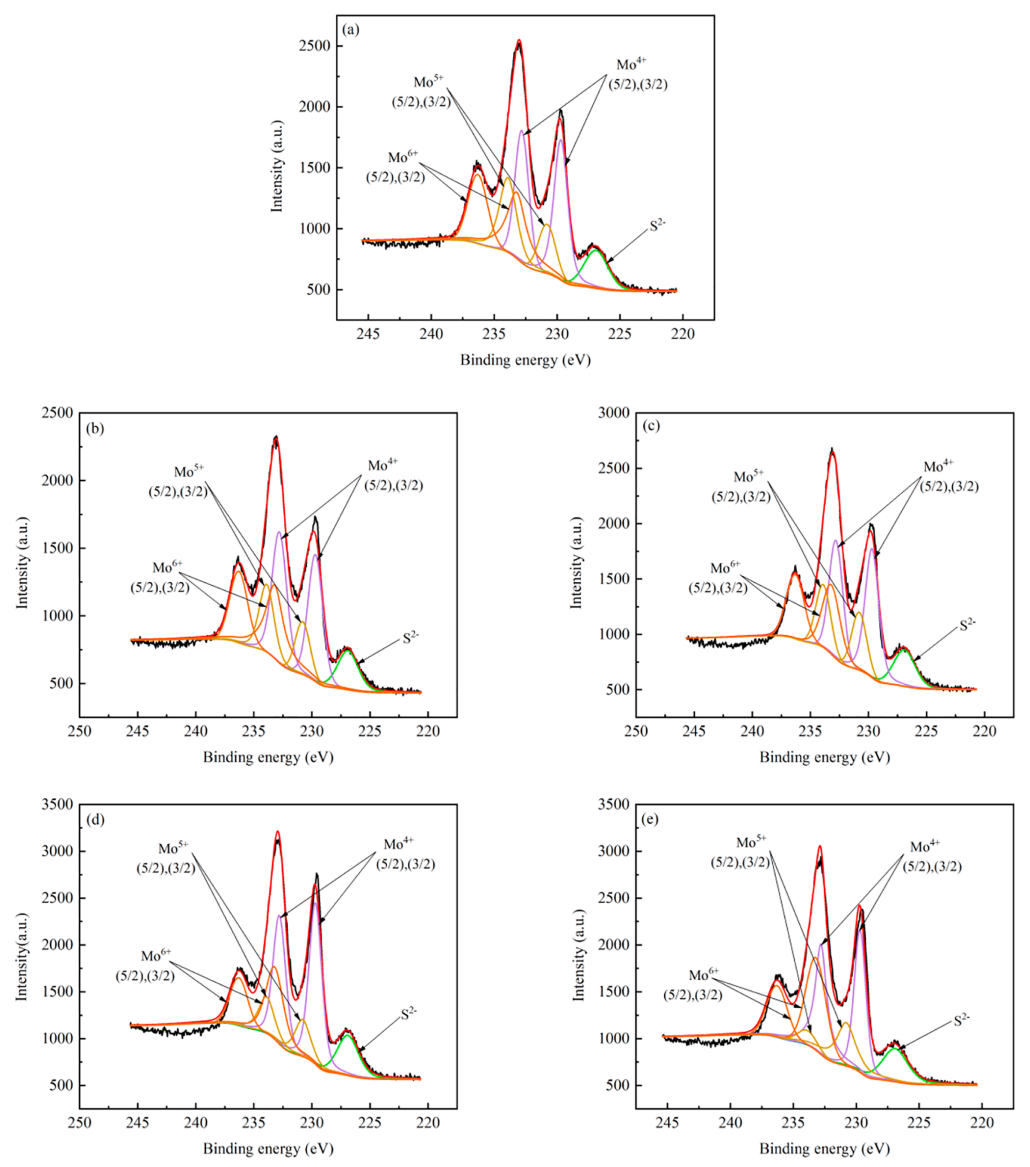
XPS spectra (Mo 3d) of MoS_2_/AC catalysts: (a) MoS_2_/AC, (b) MoS_2_/AC1, (c) MoS_2_/AC2, (d) MoS_2_/AC3, (e) MoS_2_/AC4.

The distribution of metal species (Mo) with different valence states in the MoS_2_/AC catalysts has been characterized by XPS, and the result is shown in Figure 6. Figure 6 displays the Mo 3d spectra of the MoS_2_/AC series catalysts including three kinds of Mo species (Mo^4+^, Mo^5+^, Mo^6+^). The binding energies appearing at 229.7 and 232.8 eV are relevant to the Mo^4+^ 3d_5/2_ and 3d_3/2_. And the tetravalent Mo ions mainly existing in the form of fully sulfurized MoS_2_, which is closely related with the naphthalene hydrogenation activity.^30,31^ The binding energies for the Mo 3d_5/2_ and 3d_3/2_ of Mo^5+^ corresponding to the incompletely sulfurized MoS_*x*_O_*y*_ intermediates are located at 230.8 and 233.9 eV. The binding energies of Mo^6+^ 3d_5/2_ and 3d_3/2_ appearing at 233.2 and 236.3 eV correspond to the unsulfurized MoO_3_.^32−34^ Furthermore, the signal ascribed to S^2–^ is about at 226.9 eV.

The detailed fitting data of three valence Mo species over the catalysts are summarized in Table 5 and define the proportion of Mo^4+^ to all of the Mo species as the sulfurization degree.^35^ It can be seen from Table 5 that the proportion of Mo^4+^ is the largest among all three Mo species, indicating that the sulfurization effect of the catalysts is achieved. The sulfurization degree is related to the dispersion of the active components on the support. The modification of AC with proper concentration HNO_3_ treatment is beneficial to the dispersion of the active components on the AC, thus promoting the sulfurization effect of the active components, forming active MoS_2_ phase, and improving the hydrogenation activity of the catalyst. It can be clearly seen from Table 5 that the order of sulfurization degree of the catalysts is as follows: MoS_2_/AC3 (52.13%) > MoS_2_/AC2 (46.2%) > MoS_2_/AC4 (45.72%) > MoS_2_/AC1 (44.5%) > MoS_2_/AC (42.86%). Note that the sulfurization degree of MoS_2_/AC3 catalyst is the highest among the catalysts of ACx, which is the result of modification of AC with proper concentration of HNO_3_. Meanwhile, it can be predicted that the catalyst of MoS_2_/AC3 will have good catalytic performance. Generally, the Mo sulfurization degree on AC3 in this study is lower than that on ZrKT-60 or other support,^35^ which may be related to the presence of many micropores of AC. It is reported that micropores on carrier can prevent the full sulfurization of MoO_3_ to a certain extent.^36^

**Table 5:** Mo 3d Fitting Data of MoS_2_/AC Catalysts

catalysts	Mo^4+^,^a^ %	Mo^5+^,^b^ %	Mo^6+^,^c^ %	S_Mo_,^d^ %	MoS_2_,^e^wt %
MoS_2_/AC	42.86	24.24	32.90	42.86	8.57
MoS_2_/AC1	44.50	20.82	34.68	44.50	8.90
MoS_2_/AC2	46.20	22.65	31.15	46.20	9.24
MoS_2_/AC3	52.13	18.14	29.73	52.13	10.42
MoS_2_/AC4	45.72	18.20	36.08	45.72	9.14

a Percentage of XPS peak area of fully sulfurized MoS_2_.

b Percentage of XPS peak area of incompletely sulfurized MoS_*x*_O_*y*_.

c Percentage of XPS peak area of unsulfurized MoO_3_.

d Sulfurization degree S_Mo_ = Mo^4+^/ (Mo^4+^ + Mo^5+^ + Mo^6+^).^35^

e Amount of MoS_2_, MoS_2_ = S_Mo_ × 20% (the amount of MoO_3_).

### 3.3 Catalytic Hydrogenation of Naphthalene

#### 3.3.1 Hydrogenation of Naphthalene over the Catalysts

**Figure 7:**
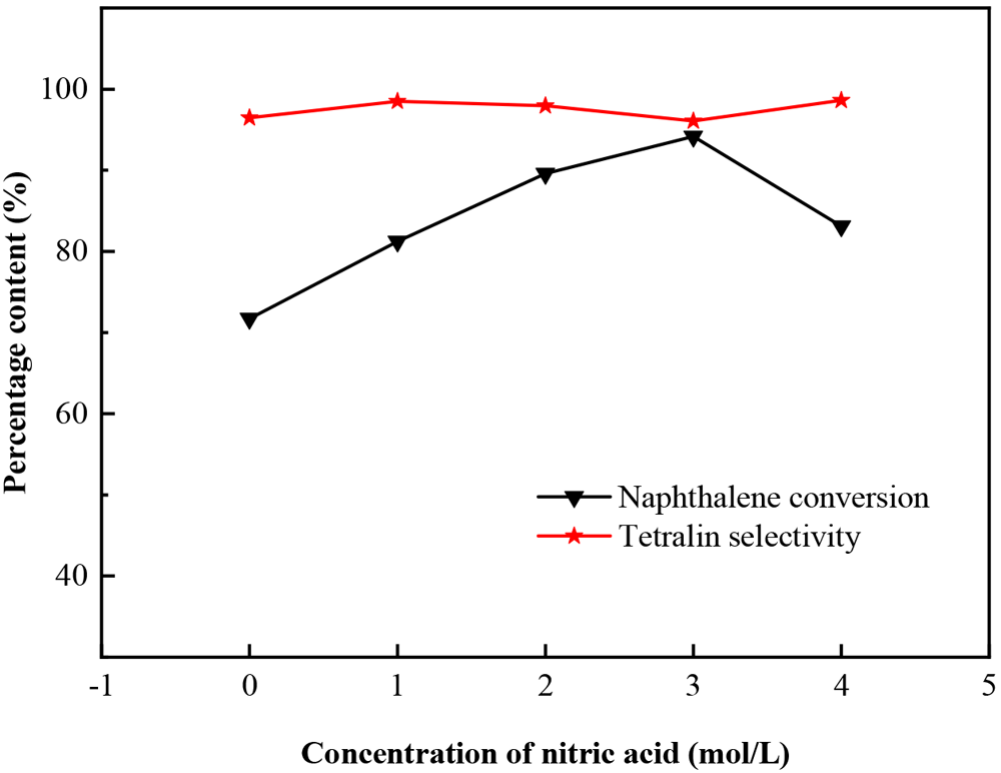
Effects of HNO_3_ concentration on naphthalene conversion and tetralin selectivity.

The naphthalene conversion and tetralin selectivity over the catalysts are shown in Figure 7. It shows that the naphthalene conversion of the catalyst with AC1–AC4 is higher than that of the catalyst with AC (MoS_2_/AC), which is attributed to the introduction of oxygen-containing functional groups on the surface of AC1–AC4. As shown in Table 4, the number of oxygen-containing functional groups on the surface of activated carbon increases as the HNO_3_ concentration used increases. The oxygen-containing functional group can improve the hydrophilicity of the surface of activated carbon, which is beneficial to the metal precursor solution entering the pore of the AC during the impregnation process, thus making the metal dispersion more uniform. At the same time, the oxygen-containing functional group can also be used as the anchor of active metal to improve the metal dispersion.^19,22^ The good dispersion of active metal can improve the catalytic performance of the catalyst.^19,37^ It should be noted that the conversion of naphthalene increases first and then decreases as the HNO_3_ concentration increases, and the naphthalene conversion over the catalyst of MoS_2_/AC3 is the highest. However, the naphthalene conversion over the catalyst of MoS_2_/AC4 decreased, indicating that a too high concentration of HNO_3_ treatment of AC is not conducive to naphthalene hydrogenation. It may be since too many oxygen-containing groups on the surface of AC can limit the anchoring of active metal, thus affecting the dispersion of active components.^19,22^ Also, the results of STEM characterization confirm that the dispersion of MoS_2_/AC4 catalyst was lower than that of MoS_2_/AC3. This is consistent with the results of Li et al, who studied the Pd catalyst supported on the AC modified with HNO_3_ for nitrobenzene hydrogenation.^20^ For tetralin selectivity, both MoS_2_/AC and MoS_2_/AC1–AC4 catalysts can get more than 96%. It indicates that HNO_3_ treatment of AC has little effect on the selectivity of tetralin. All the higher selectivity of tetralin is due to the proper hydrogenation ability of MoS_2_, which can avoid the saturation of tetralin to produce decalin. At the same time, the lack of strong acid sites in the catalyst can reduce cracking of hydrogenation products. Consequently, a higher than 96% of tetralin selectivity has obtained over untreated and modified catalyst.

#### 3.3.2 Effect of Temperature on Hydrogenation of Naphthalene

**Figure 8:**
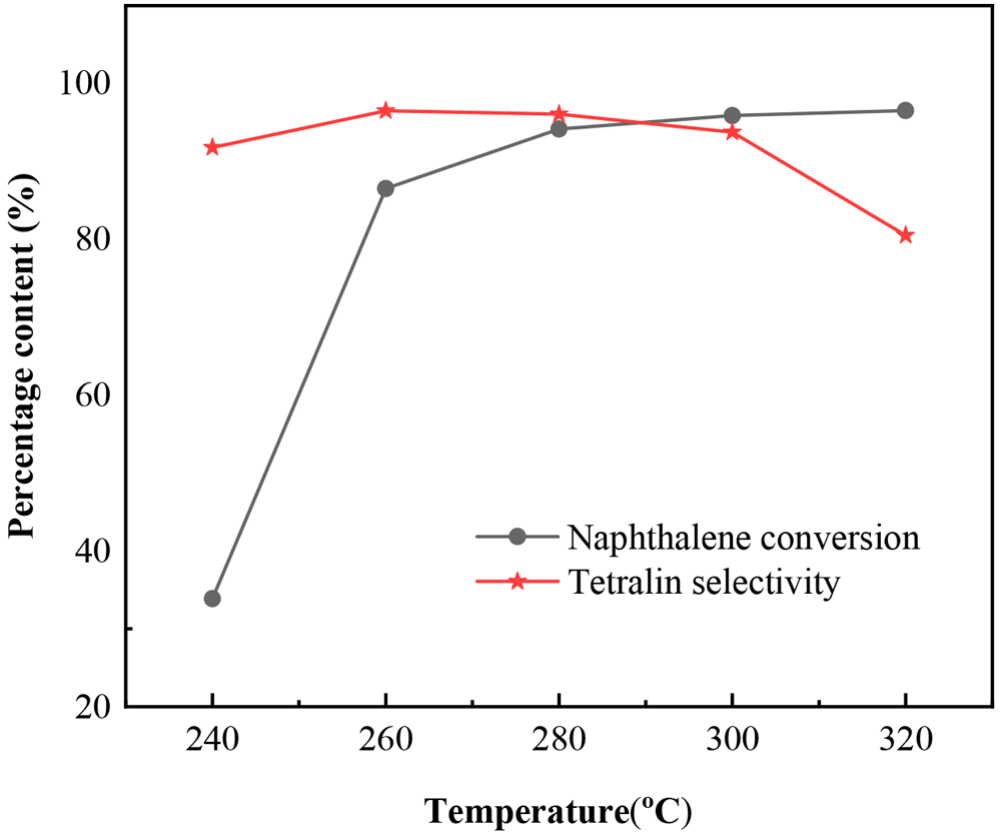
Effect of temperature on naphthalene conversion and tetralin selectivity over MoS_2_/AC3 catalyst (reaction conditions: *P* = 4 MPa, LHSV = 2 h^–1^, H_2_/oil = 1000).

As mentioned above, MoS_2_/AC3 catalyst shows good catalytic performance. Therefore, it is an example to study the selective catalytic hydrogenation of naphthalene to tetralin at different temperatures (240, 260, 280, 300, 320 °C). The results are shown in Figure 8. It can be seen that the conversion of naphthalene increases as the reaction temperature increases, since the increase of temperature is beneficial to improve the hydrogenation reaction rate according to reaction kinetics.^14^ The selectivity of tetralin almost keeps constant at 260–300 °C and then decreases obviously at 320 °C. This is possibly caused by the hydrogenation of naphthalene to tetralin being an exothermic reaction, which is disadvantageous at high temperature, and thus, the selectivity of tetralin decreases.^14^ It can also be calculated that the yield of tetralin is the highest at 280 °C from Figure 8. Therefore, 280 °C is the optimal temperature for selective hydrogenation of naphthalene to tetralin over the MoS_2_/AC3 catalyst.

#### 3.3.3 Comparison of MoS_2_/AC3 and MoS_2_/Al_2_O_3_ Catalysts

**Figure 9:**
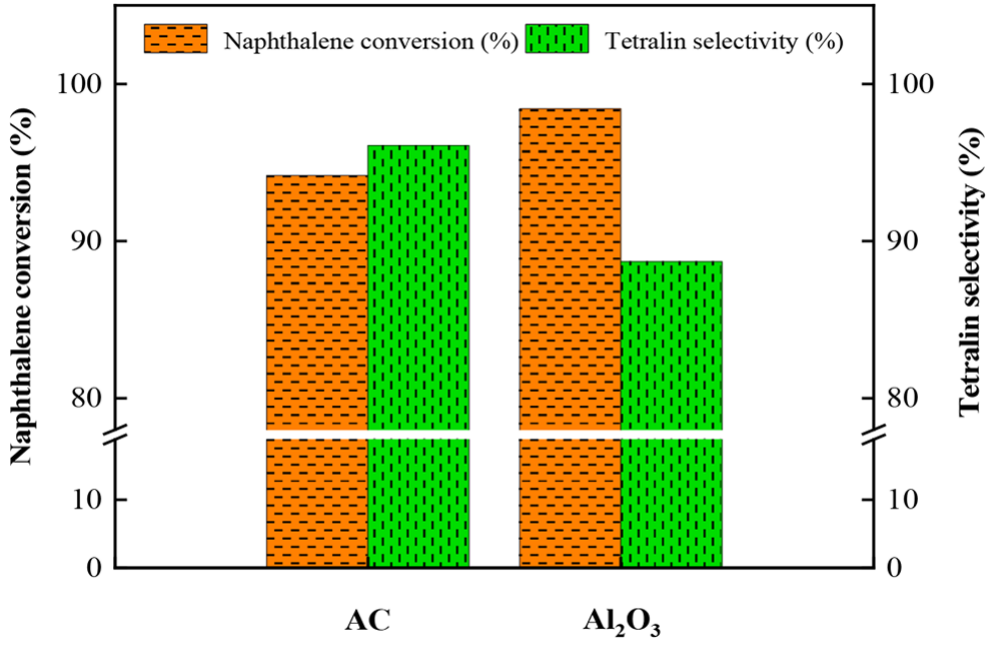
Naphthalene conversion and tetralin selectivity of MoS_2_/AC3 and MoS_2_/Al_2_O_3_ catalysts (reaction conditions: *T* = 280 °C, *P* = 4 MPa, LHSV = 2 h^–1^, H_2_/oil = 1000).

Generally, Al_2_O_3_ has been commonly used as the support of naphthalene hydrogenation catalyst.^12^ In order to compare the catalytic performance of the catalysts of MoS_2_/AC3 with that of prepared by conventional Al_2_O_3_, the MoS_2_/Al_2_O_3_ catalyst was also tested for naphthalene hydrogenation under the same conditions and the result is shown in Figure 9. It can be seen from Figure 9 that the naphthalene conversion of MoS_2_/Al_2_O_3_ is slightly higher than that of the MoS_2_/AC3 catalyst and the selectivity of tetralin is significantly lower than that of the MoS_2_/AC3 catalyst. The high naphthalene conversion of MoS_2_/Al_2_O_3_ catalyst is attributed to the high amount of acid site on MoS_2_/Al_2_O_3_ (in Table 2), which also can be used as the active site for naphthalene hydrogenation,^17^ thus increasing the naphthalene conversion.

In general, the hydrogenation of naphthalene is a continuous reaction. The first step of the reaction is to produce tetralin, and the second step of the reaction is to hydrogenate tetralin to decalin.^18^ The hydrogenation products can be further cracked into ethylbenzene, butyl-cyclohexane, 1-methyl-indan, methyl-cyclopentane, and so on under the strongly acidic catalysts.^18^ The low selectivity of tetralin over MoS_2_/Al_2_O_3_ catalyst may be due to the strongly acidic catalysts and more acid sites of MoS_2_/Al_2_O_3_, which catalyze hydrogenation of naphthalene to produce decalin as well as some cracking products. The hydrogenation products over MoS_2_/AC3 and MoS_2_/Al_2_O_3_ catalysts are shown in Table 6. It can be clearly seen that some cracking products of 1-methyl-indan and ethylbenzene can be obtained over the two catalysts. However, methyl-cyclopentane can be obtained over the MoS_2_/Al_2_O_3_, while it cannot over MoS_2_/AC3 catalyst, which is agreement with the report.^38^ Meanwhile, more decalin can be produced over MoS_2_/Al_2_O_3_ than that over MoS_2_/AC3 catalyst. The high performance of MoS_2_/AC catalyst also may be attributed to the micropores of activated carbon, which is conducive to the naphthalene hydrogenation to tetralin, thus increasing the selectivity of tetralin.^36^ Therefore, MoS_2_/AC3 catalyst is suitable for the hydrogenation reaction of naphthalene to tetralin.

**Table 6:** Product Distribution over MoS_2_/AC3 and MoS_2_/Al_2_O_3_

	product distribution (%)					
	hydrogenation saturation products			cracking products		
catalysts	tetralin	trans-decalin	cis-decalin	ethyl-benzene	1-methyl-indan	methyl-cyclopentane
MoS_2_/AC3	96.07	2.33	1.02	0.34	0.24	0
MoS_2_/Al_2_O_3_	88.75	7.34	3.17	0.20	0.27	0.27

#### 3.3.4 Mechanism of Hydrogenation of Naphthalene

In previous studies, Liu et al. discussed the reaction of naphthalene on Mo-based catalysts according to two reaction mechanisms. The first is the monomolecular mechanism, which is that naphthalene is hydrogenated to tetralin and decalin at the active site, and then the hydrogenation product migrates to the acid site, which is further hydrocracked to ring-opening product through the overflow hydrogen from the active site. However, when the available activated hydrogen supply to the acid site is insufficient, the reaction follows the bimolecular mechanism, which is that a tetralin molecule forms carbenium ion of alkylbenzene at the acid site and then reacts with naphthalene to form alkylnaphthalene and multinuclear aromatics.^37^ In this study, the product distribution on the catalysts (see Table 6) shows that the main product includes tetralin, trans-decalin, cis-decalin, 1-methyl-indan, methyl-cyclopentane, and ethylbenzene, indicating that the reaction mechanism follows monomolecular mechanism because of sufficient available activated hydrogen supply.

**Figure 10:**
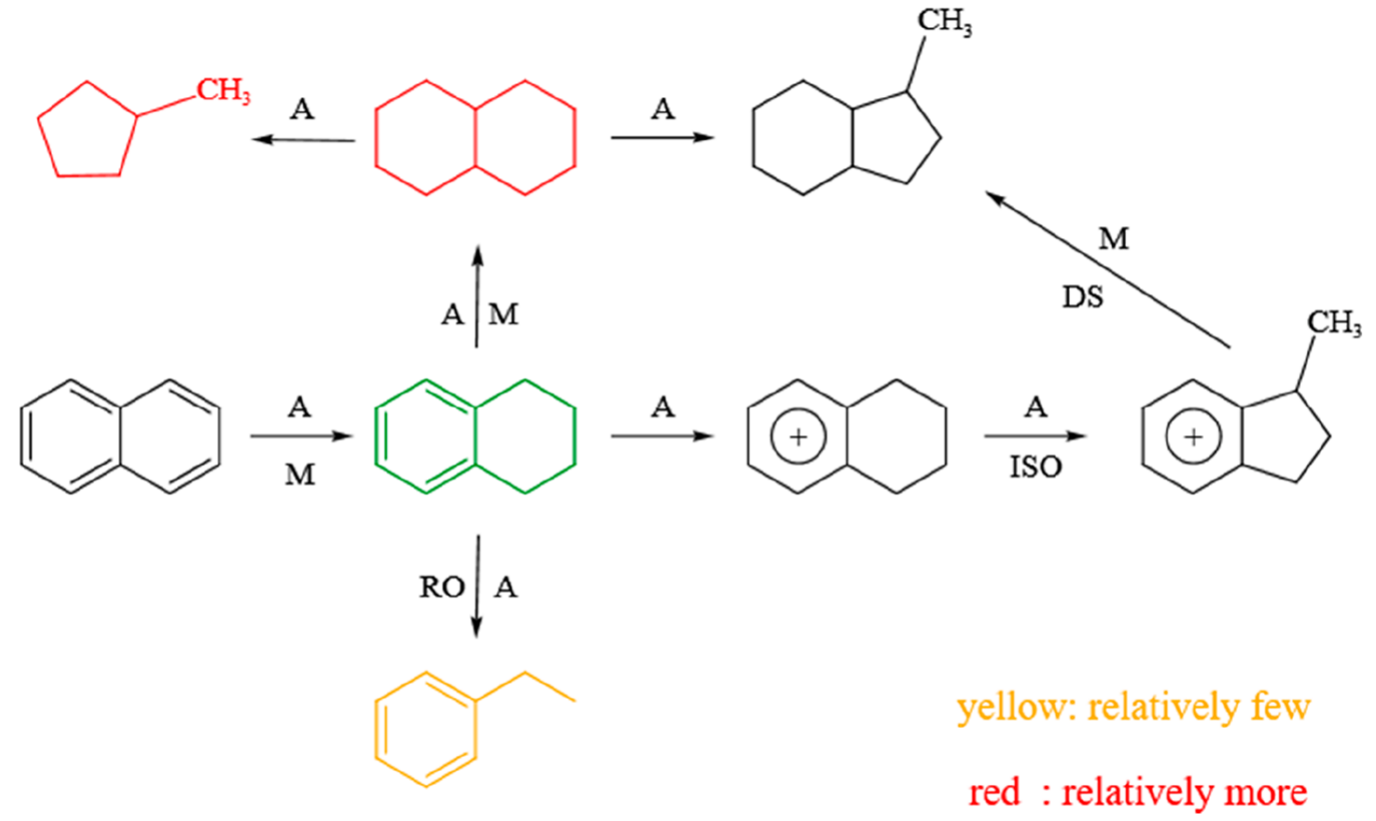
Reaction path of naphthalene on MoS_2_/ Al_2_O_3_ catalyst. A, acid sites; M, metal sites; ISO, isomerization; DS, desorption; RO, ring opening.

Figure 10 shows the reaction path of naphthalene under MoS_2_/Al_2_O_3_ catalyst. Naphthalene produces tetralin under the combined action of acid site and MoS_2_ active site.^38,39^ Tetralin forms carbenium ion of alkylbenzene at the acid site, then isomerizes to methyl-indene carbocation, and finally desorbed to 1-methyl-indan at the active site. When tetralin is affected by overflow hydrogen at the acid site, it can also break the α and γ sites of benzene ring to form ethylbenzene. At the same time, tetralin can also produce decalin under the combined action of acid site and MoS_2_ active site. Decalin is further hydrocracked to 1-methyl-indan and methyl-cyclopentane at the acid site by overflowing hydrogen from the active site of MoS_2_.

**Figure 11:**
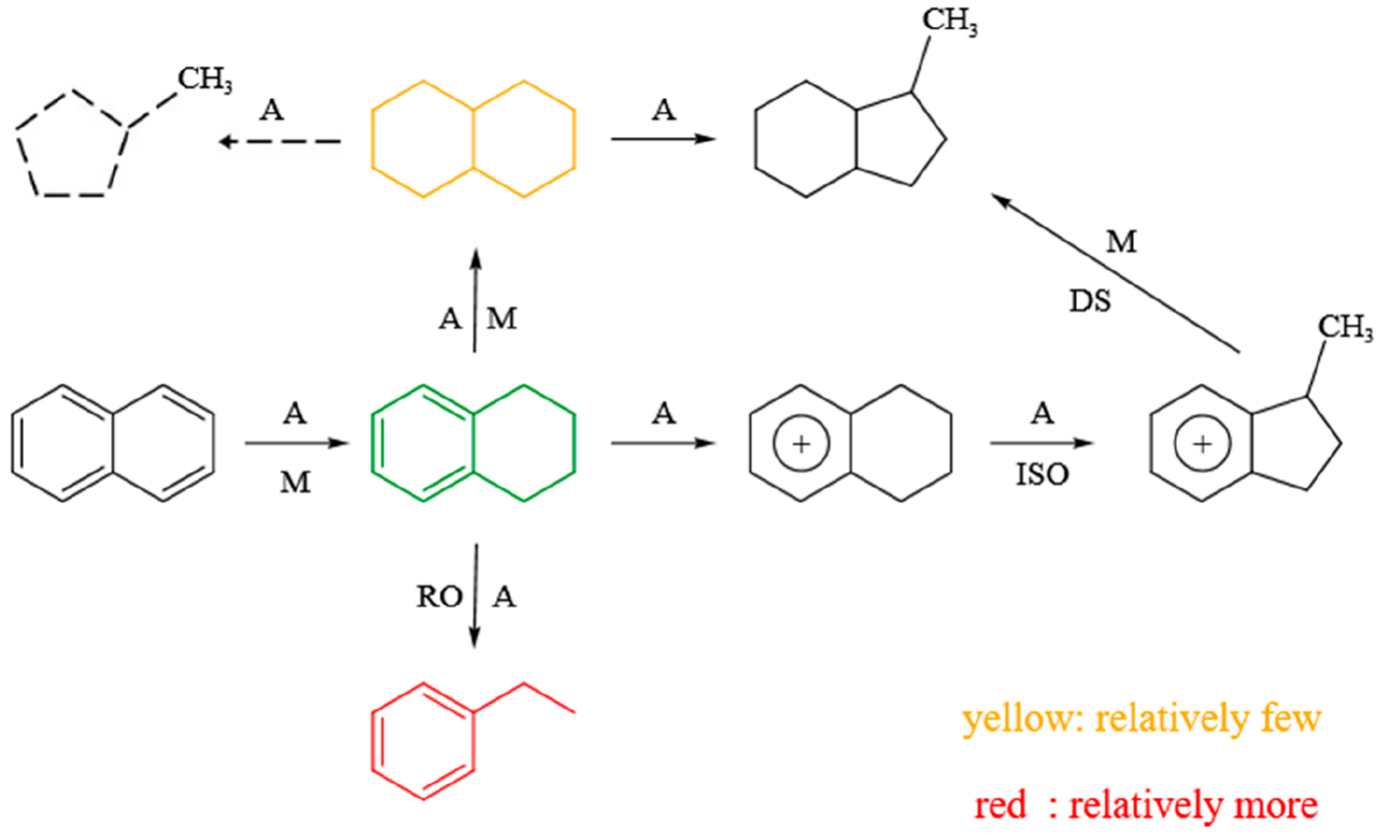
Reaction path of naphthalene on MoS_2_/AC catalyst. A, acid sites; M, metal sites; ISO, isomerization; DS, desorption; RO, ring opening.

The hydrogenation path of naphthalene over MoS_2_/AC catalyst is shown in Figure 11. Similar with that over the MoS_2_/Al_2_O_3_ catalyst, naphthalene over the MoS_2_/AC catalyst can produce tetralin and decalin under the combined action of acid site and MoS_2_ active site. Tetralin forms carbenium ion of alkylbenzene at the acid site, then isomerizes to methyl-indene carbocation, and finally desorbed to 1-methyl-indan at the active site. At the same time, when tetralin is affected by overflow hydrogen at the acid site, the α and γ sites of benzene ring are broken to form ethylbenzene. Decalin is further hydrocracked to 1-methyl-indan by overflowing hydrogen from the MoS_2_ active site on the acid site. Compared with MoS_2_/Al_2_O_3_ catalyst, the MoS_2_/AC catalyst produces more tetralin and less decalin, which is because the MoS_2_/AC catalyst has fewer acid sites than the MoS_2_/Al_2_O_3_ catalyst. The acid sites also can be used as the active site for naphthalene hydrogenation,^17^ which is favorable for further reaction of tetralin to produce decalin. Meanwhile, the ethylbenzene produced by tetralin cracking over the MoS_2_/AC catalyst is slightly higher than that over the MoS_2_/Al_2_O_3_ catalyst, which is the result of more tetralin produced under the MoS_2_/AC catalyst and then tetralin has cracked into ethylbenzene. Moreover, methyl-cyclopentane is not found over the MoS_2_/AC catalyst, indicating that decalin is not cracked into methyl-cyclopentane, which is possibly the result of the absence of strong acid sites over the MoS_2_/AC catalyst. Therefore, the MoS_2_/AC catalyst shows a high yield of tetralin. Overall, the good catalytic performance of the MoS_2_/ACx catalyst for hydrogenation of naphthalene to tetralin is attributed to the large specific surface area of ACx, the suitable pore structure of the ACx, the appropriate acidity of the catalyst and the high dispersion of active components on the ACx.

## 4 Conclusion

The catalysts with high activity and yield of tetralin of MoS_2_ supported on AC1–AC4 have been successfully prepared. According to the characterization and hydrogenation results of the catalysts, the following conclusions can be drawn.

Compared with AC, the catalyst prepared by AC1–AC4 has higher naphthalene conversion and tetralin selectivity. It has proved that the modification of activated carbon with HNO_3_ is an effective way to improve the performance of the catalyst, which mainly increased the micropore surface area of AC, the micropores volume of AC, and the oxygen-containing functional groups on the surface of activated carbon. A concentration of 3 mol/L HNO_3_ is more suitable for the treatment of AC, which is more conducive to the dispersion of active metals on the AC.

Among the catalysts of AC1–AC4, the MoS_2_/AC3 has the highest naphthalene conversion and tetralin yield, which presents 94.2% naphthalene conversion and 90.5% tetralin yield under mild conditions of 280 °C and 4 MPa. This catalyst is also superior to the MoS_2_/Al_2_O_3_ for the yield of tetralin because of its large specific surface area and high MoS_2_ dispersion as well as the absence of strong acid sites on the catalyst. It is suitable for the hydrogenation reaction of naphthalene to tetralin.
